# Bibliographical Notices

**Published:** 1856-04

**Authors:** 


### EDITORIAL DEPARTMENT.

### BIBLIOGRAPHICAL NOTICES.

*An Essay on Intermittent and Bilious Remittent Fevers: with their Pathological relation to Ozone.* By E. S. Gaillard, M. D., Charleston. Walker & Evans, 1856.

This little treatise was originally submitted to the faculty of the Medical College of South Carolina, as an inaugural dissertation, for the degree of doctor of medicine. By them it was considered worthy of the annual premium bestowed by that college upon the best essay submitted by the graduating class. By the author it is so favorably thought of that he has given it to the public in its present form. We doubt the propriety of this. It is indeed highly creditable as a thesis of a candidate for graduation, but it appears to us that its value would have been increased had its author delayed its publication till matured experience had corrected or confirmed some of the hypotheses advanced. This would have enabled him to avoid some grave errors.

In speaking of ptyalism, for example, he has wholly misconceived Melseus' views of the application of iodide of potassium to the treatment of mercurial poisoning. Dr. Gaillard recommends its use in cases of *dangerous salivation.* The only effect it could have, according to the views of the European chemist, under such circumstances, would be to increase the mischief, for, if any insoluble compounds were deposited in the system, they would be at liberty by the salt and would be equivalent to so many additional fresh doses of mercury. It is familiar to every one that after a protracted course of mercurials, say in a case of syphilis, for example, the administration of iodide of potassium suddenly produces the salivation which the mercury had failed to accomplish. It could hardly act in this manner, except by setting at liberty the insoluble compound of quicksilver which had accumulated in the system.

*A Paper on the Effects of Lead on the Heart.* By John W. Carson, M. D., New York, 1856.

This very valuable paper contains the history of ten cases of lead poisoning, studied with especial reference to the influence of that cachexy upon the heart. The action is that of a sedative, as shown by diminution of the force of the impulse, faintness upon exertion, occasional attacks of alarming syncope sinking palpitation, and a slow, soft, compressible pulse. We commend the paper to the careful perusal of all interested in the study of lead poisoning.

*Narrative of a Journey to the Summit of Mont Blanc.* By Wm. Howard, M. D. Baltimore, Lucas Brothers, 1856.

Dr. Howard, a gentleman highly esteemed in this his native city, was one of the few travellers who have ever stood upon the summit of Mont Blanc. The mob of tourists who prattle about ascending this "monarch of mountains," halt far below the top. The first real ascent was made by Jacques Balmut and Dr. Paccard, of Chamouni, in 1786. Dr. Saussure was the next who accomplished this feat, and he published a glowing account of the ascent. After this, several parties made this difficult and dangerous trip. Drs. Howard and Van Rensselaer were the first Americans who ever reached the summit of this famous mountain. They went up together in July, 1819, and the book before us is a graphic and entertaining account of their expediiion. They suffered, as all tourists, who attempt to climb Mont Blanc, suffer. Their eyes were inflamed, their faces blistered.

